# Matriptase Complexes and Prostasin Complexes with HAI-1 and HAI-2 in Human Milk: Significant Proteolysis in Lactation

**DOI:** 10.1371/journal.pone.0152904

**Published:** 2016-04-04

**Authors:** Chih-Hsin Lai, Ying-Jung J. Lai, Feng-Pai Chou, Hsiang-Hua D. Chang, Chun-Che Tseng, Michael D. Johnson, Jehng-Kang Wang, Chen-Yong Lin

**Affiliations:** 1 Department of Dentistry Renai Branch, Taipei City Hospital, Taipei, Taiwan; 2 Graduate Institute of Medical Sciences National Defense Medical Center, Taipei, Taiwan; 3 Department of Oncology, Lombardi Comprehensive Cancer Center, Georgetown University, Washington DC, United States of America; 4 Department of Biochemistry, National Defense Medical Center, Taipei, Taiwan; Florida State University, UNITED STATES

## Abstract

Significant proteolysis may occur during milk synthesis and secretion, as evidenced by the presence of protease-protease inhibitor complex containing the activated form of the type 2 transmembrane serine protease matriptase and the transmembrane Kunitz-type serine protease inhibitor HAI-1. In order to identify other proteolysis events that may occur during lactation, human milk was analyzed for species containing HAI-1 and HAI-2 which is closely related to HAI-1. In addition to the previously demonstrated matriptase-HAI-1 complex, HAI-1 was also detected in complex with prostasin, a glycosylphosphatidylinositol (GPI)-anchored serine protease. HAI-2 was also detected in complexes, the majority of which appear to be part of higher-order complexes, which do not bind to ionic exchange columns or immunoaffinity columns, suggesting that HAI-2 and its target proteases may be incorporated into special protein structures during lactation. The small proportion HAI-2 species that could be purified contain matriptase or prostasin. Human mammary epithelial cells are the likely cellular sources for these HAI-1 and HAI-2 complexes with matriptase and prostasin given that these protease-inhibitor complexes with the exception of prostasin-HAI-2 complex were detected in milk-derived mammary epithelial cells. The presence of these protease-inhibitor complexes in human milk provides *in vivo* evidence that the proteolytic activity of matriptase and prostasin are significantly elevated at least during lactation, and possibly contribute to the process of lactation, and that they are under tight control by HAI-1 and HAI-2.

## Introduction

Proteolysis, a process involving the cleavage of peptide bonds by hydrolytic enzymes proteases, has been implicated in many physiological and pathologic processes. Although specific roles for proteases in lactation have largely remained ill-defined and poorly characterized, it is not unreasonable to suppose that proteases participate in many cellular and molecular processes involved in milk synthesis and secretion. Many proteases are synthesized as zymogens and acquire enzymatic activity only after cleavage at a canonical activation motif, which is an irreversible process. Furthermore, once activated the hydrolytic activity of active proteases could cause tremendous damages if left unchecked. One of the mechanisms that prevent undesired proteolysis is the action of protease inhibitors, a class of proteins which can bind to and inactivate active proteases. The presence of activated proteases in complexes with their cognate inhibitors in milk is, therefore, strongly suggestive that the proteases in question have been activated and have participated in some ways during the course of milk synthesis and secretion. For example, the type 2 transmembrane serine protease matriptase was purified from human milk in its activated form in complex with its cognate inhibitor, hepatocyte growth factor (HGF) activator inhibitor (HAI)-1 [1]. The presence of considerable amounts of activated matriptase in human milk suggests that matriptase proteolytic activity is significantly induced during lactation. That this activated matriptase is secreted as a complex with HAI-1 further indicates that matriptase proteolytic activity is under the tight control of this integral membrane Kunitz type serine protease inhibitor.

Both matriptase and HAI-1 are widely expressed by the epithelial components of most organ systems, including the mammary gland [2,3]. While the exact role of matriptase in lactation remains elusive, studies of human genetic disorders that dysregulated matriptase expression or function and data from mouse models with targeted deletion of matriptase suggest that the enzyme is required for epidermal barrier function and the maintenance of epithelial integrity and function [4,5]. Many downstream matriptase substrates have been identified including the epithelial sodium channel (ENaC) [6], the GPI-anchored serine protease prostasin [7], the major extracellular matrix-degrading protease urokinase type plasminogen activator (uPA) [8,9], the G protein-coupled receptor protease activated receptor (PAR) 2 [9], the growth/motility factor hepatocyte growth factor (HGF) [8], and platelet-derived growth factor (PDGF) D [10]. Matriptase may contribute to lactation through some of these known substrates or through other substrates that have yet to be identified.

In addition to HAI-1, matriptase can also be inactivated by other serine protease inhibitors. The control of matriptase by antithrombin, a blood-borne secreted serpin, was initially recognized by identification and purification of matriptase-antithrombin complexes from human milk [11]. In human keratinocytes, the levels of membrane-associated antithrombin can inversely affect the level of free active matriptase shed to the extracellular milieu [12]. More recently, HAI-2, a Kunitz inhibitor that is highly related to HAI-1, has been shown to also be a matriptase inhibitor in breast cancer cells, but not in cultured mammary epithelial cells [13,14]. The differential role of HAI-2 in matriptase inhibition results from a proportion of HAI-2 being targeted to the surface of breast cancer cells, whereas the vast majority of HAI-2 remains on the inside of mammary epithelial cells. Given that HAI-1 and HAI-2 are expressed at high levels by mammary epithelial cells, we hypothesize that the target proteases of both Kunitz inhibitors might be actively involved in lactation, and if so, having subsequently been inactivated by forming complexes with HAI-1 and HAI-2 and secreted they should be detectable in the milk. In the current study we, therefore, set out to identify and characterize HAI-1 and HAI-2 complexes present in human milk. Our purification scheme and subsequent protein identification work reveal that in addition to matriptase-HAI-1 complexes, human milk contains matriptase-HAI-2, protsatin-HAI-1 and prostasin-HAI-2 complexes. Our study suggests that matriptase and prostasin proteolytic activity is significant during lactation and that these activities are tightly regulated by HAI-1 and HAI-2.

## Materials and Methods

### Chemicals and reagents

The conventional liquid chromatography resins CM-Sepharose and DEAE-Sepharose, and activated Sepharose beads were obtained from GE healthcare, (Piscataway, NJ). Protoblue Safe was purchased from National Diagnostics (Atlanta, Georgia).

### Cell cultures

The milk-derived MTSV-1.7 human mammary epithelial cells, a gift from Dr. J. Taylor-Papadimitriou (Imperial Cancer Research Fund, London) [15], were cultured in a modified Improved Minimum Essential Medium (IMEM), supplemented with 10% FBS.

### Monoclonal antibodies (mAbs)

Monoclonal antibodies (mAbs) against human matriptase (M24), HAI-1 (M19), and HAI-2 (DC16) were used for immunoblot analysis as previously described [13,14]. The anti prostasin mAbs, YL10, YL11, and YL89 were generated by conventional hybridoma fusion using the purified prostasin-HAI-1 complexes as the antigen. The validation and characterization of these prostasin antibodies is described below.

### Immobilization of mAbs

The mAbs 21–9, M19, DC16, and YL11 were covalently coupled to Sepharose 4 B at 5mg/ml gel following the manufacturer’s instructions (GE healthcare, Piscataway, NJ). Briefly, the mAbs were purified and dialyzed against 0.1 M sodium bicarbonate containing 0.5 M sodium chloride and then mixed and incubated with the CNBr-activated Sepharose 4B on a rotator overnight in the cold room. Any uncoupled mAb was removed by washing the beads with coupling buffer, after which the residual coupling sites on beads were blocked by 1 M Tris buffer.

### CM-Sepharose and DEAE-Sepharose chromatography

Expired human milk was obtained from Georgetown University Medical Center Milk Bank. The milk was thawed and centrifuged to remove the milk fat and insoluble debris. The defatted milk was dialyzed against 10 mM phosphate buffer, pH 6.0. The milk (approximately 200 ml) was applied onto a CM-Sepharose FF column (2.5x20cm; GE health Science), equilibrated with 10 mM phosphate buffer, pH 6.0. The CM column was then washed with at least 1 liter of phosphate buffer, pH 6.0. Proteins were eluted with a linear gradient of 0–0.5 M NaCl in 10 mM phosphate buffer, pH 6.0, with a total volume of 500 ml.

For DEAE chromatography, the flow-through fraction from the CM-Sepharose was dialyzed against 20 mM Tris buffer pH 8.0 at 4°C and then loaded onto a DEAE-Sepharose FF column (2.5x20cm; GE health Science). After the column was washed, the bound proteins were eluted with a linear gradient of 0–1 M NaCl in Tris buffer pH 8.0, with a total volume of 500 ml.

### Immunoaffinity Chromatography

**Fig 1:**
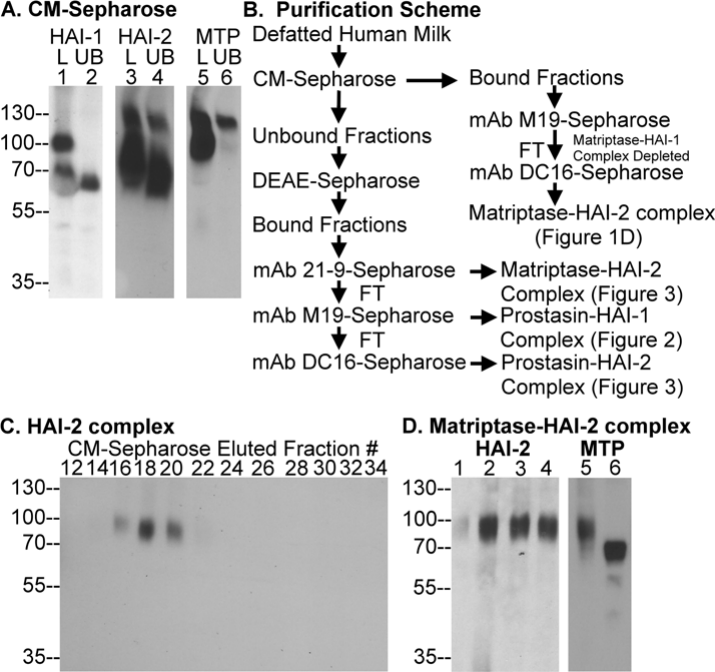
HAI-1 and HAI-2 complexes in human milk. (A) Defatted human milk was loaded onto a CM-Sepharose column and the unbound fraction (UB) was collected. The milk (lanes 1) and the unbound fraction (lanes 2) were analyzed by immunoblot for HAI-1, HAI-2, and matriptase (MTP), as indicated. (B) Flow-chart summarizing the purification scheme for the HAI-1 and HAI-2 complexes in human milk. (C) Fractions 12 through 34 eluted from the CM-Sepharose chromatography were analyzed by Western blot for HAI-2. (D) The pooled HAI-2 containing fractions eluted from the CM-Sepharose were subjected to immunoaffinity chromatography on a HAI-1 mAb M19-Sepharose column followed by a HAI-2 mAb DC16-Sepharos column. Fractions 1 through 4 eluted from the mAb DC16-Sepharose were analyzed by Western blot for HAI-2 species (left panel). The pooled HAI-2 containing fractions were analyzed by immunoblot for matriptase species under non-boiled (lane 1) and boiled (lane 2) conditions (right panel).

Three immunoaffinity columns were used: matriptase mAb 21-9-Sepharose, HAI-1 mAb M19-Sepharose, and HAI-2 mAb DC16-Sepharose, each of which had a column bed volume of approximately 2 ml. Immunoaffinity chromatography was carried out with two or three columns connected in series as indicated in Fig 1. The pooled column fractions from the ionic exchangers were loaded onto the immunoaffinity columns at a flow rate of 6 ml/h. After extensive washing with phosphate buffer saline, the bound proteins were eluted from the immunoaffinity columns with 0.1 M glycine buffer, pH 2.4. The eluted fractions were immediately neutralized by the addition of 2 M Trizma base.

### Western blotting

Protein samples for Western blotting were diluted with 5X SDS sample buffer and incubated either at room temperature or at 95°C for 5 min, as indicated. The sample buffer did not contain a reducing agent. Protein samples were resolved by 7.5% SDS-PAGE, transferred to nitrocellulose membranes, and probed with the mAbs, as indicated. The binding of the primary antibody was detected using HRP conjugated secondary antibodies, and visualized using Western Lightening^®^ Chemiluminescence Reagent Plus (Perkin-Elmer, Boston, MA) and x-ray film.

### Diagonal SDS polyacrylamide gel electrophoresis (PAGE)

The purified prostasin-HAI-1 complexes from the immunoaffinity column were resolved by a 7.5% SDS-PAGE under non-boiled conditions. A gel strip was then sliced from the gel from the previous step, which was then boiled with SDS sample buffer containing no reducing agent. The boiled gel strip was placed on the top of a new 7.5% SDS-PAGE gel and subjected to electrophoresis in the second dimension. The first and second dimensional gels were stained using Protoblue Safe (National Diagnostics). The protein spots of interest were sliced from the gel and subjected to proteomics-based protein identification provided as service by ProtTech (Eagleville, PA).

### Immunodepletion

Protein samples (200 μl) were incubated with 15 μl of the indicated mAb-Sepharose beads in the cold room for 2 hours with rotation. The supernatant was then separated from the Sepharose beads by centrifugation of 1,000 RPM for 1 minute using a table top centrifuge. The supernatant was collected and subjected to Western blot analysis.

## Results

### Purification and identification of complexes of matriptase or prostasin with HAI-1 or HAI-2 from human milk

In our previous studies [1,16], HAI-1 has been identified as the prominent inhibitor of matriptase by formation of stable complexes with activated matriptase, which find their way into human body fluids including milk, semen, and urine. In addition to forming complexes with matriptase, HAI-1 can also be involved in the control of other proteases, as HAI-1-containing species, other than matriptase-HAI-1 complex, have also been detected in human semen and urine [16]. In our recent study [13], matriptase was also shown to be subject to regulation by HAI-2, which resembles HAI-1 in its overall protein domain structure, the specificity of its protease inhibitor activity, and its tissue distribution. This suggests that HAI-2 might also be involved in the inhibition of serine proteases that are secreted into body fluids, like HAI-1-protease complexes. In the current study, in order to identify the physiologically relevant target proteases of HAI-1 and HAI-2 in this context, we analyzed the composition of the species containing one of the two Kunitz type serine protease inhibitors in human milk. Western blot analysis of human milk reveals that there are two HAI-1 species (Fig 1A, lane 1) and two HAI-2 species (Fig 1A, lane 3). The 95-kDa HAI-1 complex has been previously shown to contain matriptase [1], and this was verified by immunoreactivity with a matriptase antibody (Fig 1A, lane 5). The 95-kDa matriptase-HAI-1 complex can bind to the CM-Sepharose and so disappears from the unbound fraction (Fig 1A, lanes 2 and 6). The second HAI-1 species with a size of 60-kDa did not bind to the CM-Sepharose beads and so was detected in the unbound fraction (Fig 1A, lane 2). The two HAI-2 species (Fig 1A, lane 3) did not bind to CM-Sepharose and so remained in the unbound fractions (Fig 1A, lane 4). It should be noted that there was apparently a major milk protein with a size of about 60-kDa, which distorted the migration of the 60-kDa HAI-1 complex and the 65-kDa HAI-2 complex (Fig 1A, lanes 1 and 3). This major milk protein appears to bind to CM-Sepharose and as a result, both HAI complexes were observed with sizes in the CM unbound fractions (Fig 1A, lanes 2 and 4).

In order to purify and identify these HAI-1 and HAI-2 species, we developed a purification scheme that combined conventional liquid chromatography and immunoaffinity chromatography and that is summarized in Fig 1B. While the vast majority of HAI-2 species were detected in the CM-Sepharose unbound fractions, a minor HAI-2 species appeared to be bound to and eluted from the CM-Sepharose column (Fig 1C, #16–20). We began with the characterization of the minor HAI-2 species eluted from CM-Sepharose. The pooled CM fractions containing the 90-kDa HAI-2 complex were applied to the HAI-1 mAb M19-Sepharose column to deplete the sample of the previously well-characterized 95-kDa matriptase-HAI-1 complex [1]. The 90-kDa HAI-2 complex in the unbound fraction from the mAb M19-Sepharose column was further purified using the HAI-2 mAb DC16-Sepharose column (Fig 1D, lanes 2–4). Kunitz protease inhibitors form heat-sensitive complexes with serine proteases and because matriptase has been identified as a HAI-2 target protease, we investigated whether the 90-kDa HAI-2 complex contained matriptase by Western blot analysis under both boiled and non-boiled conditions. Our analysis revealed that the 90-kDa HAI-2 complex was not only recognized by the matriptase mAb (Fig 1D, lane 5) but the complex was also dissociated by heat treatment which converts matriptase signal from the 90-kDa complex to the 70-kDa matriptase monomer (Fig 1D, lane 6). These data suggest that the minor 90-kDa HAI-2 complex contains matriptase.

**Fig 2:**
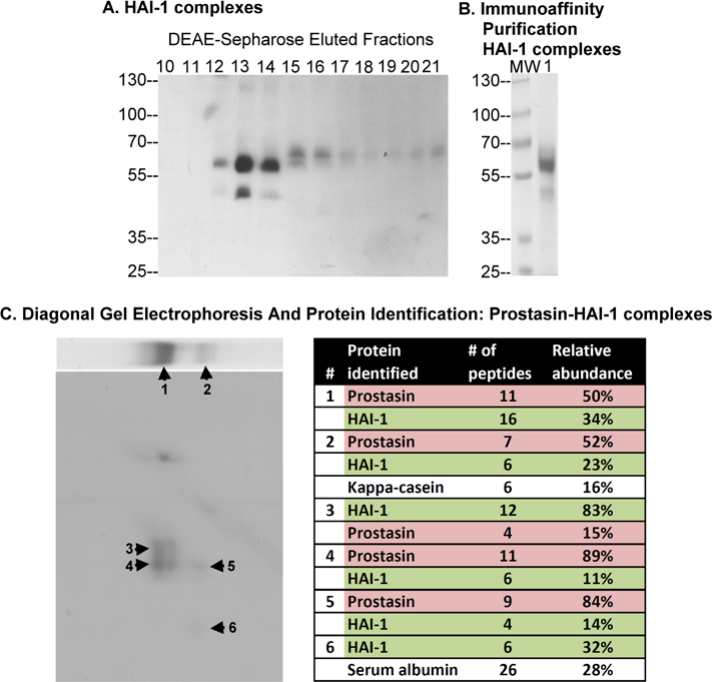
Purification and identification of prostasin-HAI-1 complex in human milk. (A) The unbound fraction from the CM-Sepharose was further purified by DEAE-Sepharose chromatography. Fractions 10–21, eluted from the DEAE-Sepharose, were analyzed by Western blot for HAI-1 species. (B) The HAI-1 containing fractions eluted from the DEAE column were subjected to immunoaffinity chromatography using HAI-1 mAb M19-Sepharose. The eluted fraction was analyzed by SDS-PAGE for the protein profile (lane 1). MW stands for molecular weight markers. (C) The HAI-1 containing species from the immunoaffinity column were analyzed by 2-dimensional diagonal gel electrophoresis (gel panels on the left) and the indicated bands subjected to MS/MS-based protein identification (table on the right). First dimension electrophoresis was carried out under non-boiled conditions (upper panel); the second dimension was carried out after heat treating the gel strip sliced from the first dimension gel (lower panel). Six protein bands, as indicated, were sliced out for protein identification. The proteins, the number of peptides, and the relative abundance identified from the 6 bands are summarized in the table on the right.

The flow-through fraction from the CM-Sepharose, which contains the 60-kDa HAI-1 complex and the vast majority of the HAI-2 complexes, was further fractionated by DEAE chromatography. Analysis of the eluted fractions by Western blot for HAI-1-containing species revealed that the 60-kDa and a minor 50-kDa species were eluted in the low salt fractions (Fig 2A, #12–14). The higher salt fractions might also contain HAI-1 species with a size slightly greater than 60-kDa (Fig 2A, #15–21). When the low salt fractions were pooled and subjected to immunoaffinity chromatography using the HAI-1 mAb M19 Sepharose, the 60- and 50-kDa HAI-1 species were purified almost to homogeneity (Fig 2B). Analysis of the HAI-1 species by diagonal gel electrophoresis revealed that the 60-kDa species could be thermally dissociated into to protein bands of 40-kDa and 30-kDa and the 50-kDa species into bands of 30-kDa and 25-kDa (Fig 2C, left panel).

When the two HAI-1-containing complexes (Fig 2C #1 and #2) and their constituent subunits (Fig 2C, #3-#6) were subjected to MS/MS-based protein identification, in addition to HAI-1, prostasin was identified being present in both complexes. The 40- (Fig 2C, #3) and 25-kDa (Fig 2C, #6) protein bands were identified as being HAI-1 and the 30-kDa band from the 60-kDa complex (Fig 2C, #4) and from the 50-kDa complex band (Fig 2C, #5) to contain prostasin. The number of tryptic peptides identified and the relative abundance of the protein identified within the 6 protein bands are summarized in the table in Fig 2C, right panel. Taken together, our purification and protein identification data reveal that the HAI-1 containing species are complexes with prostasin.

**Fig 3:**
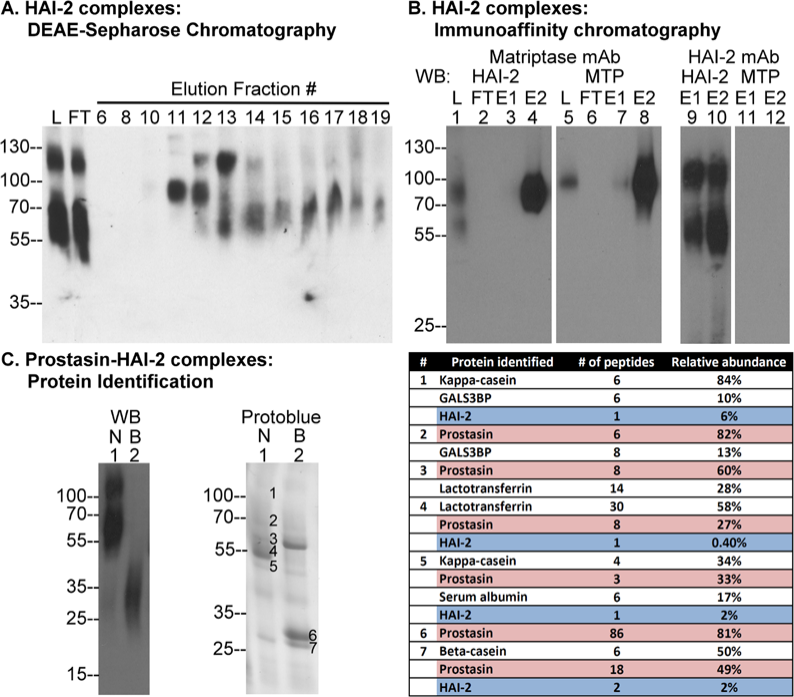
Purification and identification of HAI-2 complexes in human milk. (A) The unbound fraction from the CM-Sepharose column was subjected to DEAE-Sepharose chromatography and the bound proteins eluted by a gradient of sodium chloride. The loaded fraction (lane L), flow-through fraction (FT), and eluted fractions 6–19, were analyzed by Western blot for HAI-2 species. (B) The DEAE fractions, containing HAI-2 species, were pooled and subjected to immunoaffinity chromatography using matriptase mAb 21-9-Sepharose followed by HAI-2 mAb DC16-Sepharose. The DEAE fraction (L), flow-through (FT), and two eluted fractions (E1 and E2) from the matriptase mAb-Sepharose were analyzed by Western blot (WB) for HAI-2 species (lanes 1–4) and matriptase (MTP) species (lanes 5–8). The eluted fractions (E1 and E2) from the HAI-2 mAb-Sepharose were analyzed by Western blot for HAI-2 species (lanes 9 and 10) and matriptase (MTP) species (lanes 11 and 12). (C) The HAI-2 species purified by immunoaffinity chromatography were analyzed by Western blot (WB) for HAI-2 species and by staining with Protoblue for the protein profile under non-boiled (N) and boiled (B) conditions. Seven protein bands, as indicated, were sliced from the gel for protein identification. The proteins, the number of peptides, and the relative abundance identified from the 7 gel bands are summarized in the table on the right.

In contrast to the HAI-1 species, which was predominantly bound to DEAE Sepharose, the vast majority of the 120- and 65-kDa HAI-2 species were detected in the flow-through fraction from this column (Fig 3A, comparing FT with L; the volumes of both fractions are compatible with the FT being slightly greater). Analysis of the fractions eluted from the DEAE column reveals three HAI-2 species of 120- (#12 and 13), 90- (#11 and 12), and 65-kDa (#13-#19). It is worth noting that the volumes of eluted fractions were much smaller than that of the loading and the flow-through and so the 120- and the 65-kDa HAI-2 species in the eluted fraction represent a very small proportion of the material loaded onto the column. Similarly, the 90-kDa species also represents a minor HAI-2-containing species in human milk that can only be detected when it has been separated from the major species and concentrated by DEAE chromatography. We next pooled these DEAE fractions, and purified the HAI-2 complexes by sequential rounds of immunoaffinity chromatography as outlined in the purification scheme in Fig 1B. Given the size, the 90-kDa HAI-2 species in the pooled DEAE fraction (Fig 3B, lane 1) it seemed likely to be a HAI-2 complex with matriptase. The hypothesis was confirmed by the detection of a 90-kDa matriptase species in the pooled DEAE fraction (Fig 3B, lane 5), the capture and elution of the complex from the matriptase mAb 21-9-Sepharsoe, and the subsequent detection of this complex by both HAI-2 and matriptase mAbs (Fig 3B, lanes 4 and 8). The 120- and 65-kDa HAI-2 species were purified using the HAI-2 mAb DC-16-Sepharose (Fig 3B, lanes 9 and 10). The lack of a matriptase signal in the fractions eluted from the HAI-2 mAb DC16-Sepharsoe (Fig 3B, lanes 11 and 12) and the lack of the 120- and 65-kDa HAI-2 species in the fractions eluted from the matriptase mAb 21-9-Sepharose (Fig 3B, lanes 3 and 4) also serve to demonstrate the specificity of the immunoaffinity chromatography.

In order to identify the HAI-2 binding proteins in the 120- and 65-kDa HAI-2 complexes, the eluted fractions were concentrated and the protein profiles analyzed by SDS-PAGE and Western blot analysis under both non-boiled and boiled conditions. The heat treatment caused dissociation of the HAI-2 complexes since the HAI-2 signal was converted to its monomer size of between 25- and 35-kDa (Fig 3C, left panel, comparing lane 2 with lane 1). The heat-mediated dissociation suggests that the proteins bound to HAI-2 in these complexes could be a protease(s), as the interaction between Kunitz-type inhibitors and their target proteases are non-covalent and can be dissociated by heat treatment. The extensive N-glycan branching, which causes the diffuse banding pattern for HAI-2 and its complexes on SDS-PAGE [14] can also interfere with the staining of these proteins by protein dyes, which could result in HAI-2 and its complexes not being clearly visualized by staining with Protoblue Safe or other protein dyes. The proteins binding to HAI-2 in the complexes, however, would be more likely to run as sharp bands on SDS-PAGE and be clearly visualized by protein dyes after release from the HAI-2 complexes, assuming they are not glycosylated to same unusual extent as HAI-2. The protein profile of the purified HAI-2 species, as visualized by Protoblue Safe, appears to reflect the scenario described above. The most prominent protein band detected by Protoblue Safe staining was at 55-kDa, which is most likely the major milk protein lactotransferrin, based on the amino acid sequences obtained from the 55-kDa band (Fig 3C, #4). It remains unclear whether lactotransferrin could bind to HAI-2 in solution. Alternatively, the presence of lactotransferrin in the eluted fraction could simply result from its abundance and was purified in a non-specific manner. Diffuse, faint staining was observed between the 70-kDa and 55-kDa markers, where the 65-kDa HAI-2 complex was expected (Fig 3C, Protoblue, lane 1). There was no staining observed for the 120-kDa complex. The heat treatment, however, seemed to reduce the intensity of the faint staining of the 65-kDa complex along with the appearance of a 30-kDa protein band (Fig 3C, Protoblue, lane 2, #6). We sliced 7 pieces of gel from the Protoblue stained gel, as indicated in Fig 3C. The proteins in these 7 gel pieces were identified by in-gel trypsin digestion followed by MS/MS-based proteomic identification. While the relative abundance of HAI-2 peptides was very low, HAI-2 was clearly present in gel bands #1, #4, #5, and #7 (Fig 3C, Table). The low abundance could again result from the extensive N-glycan branching. Gel band #6, which appeared after heat treatment, was identified as prostasin with a relative abundance of up to 81% (Fig 3C, Table). The presence of prostasin in the eluted fraction from the HAI-2 mAb immunoaffinity column suggests that prostasin is likely present in these HAI-2 complexes. Consistent with this notion, prostasin was also identified in the gel bands #2-#5 with various relative abundance from 82% in gel band #2 to 27% in gel band #4. These gel bands were at the sizes corresponding to HAI-2 complexes. Collectively, our purification and protein identification data reveal that the 65-kDa HAI-2 species is a complex of HAI-2 with prostasin. The binding proteins in the 120-kDa HAI-2 complex were currently not clearly identified.

### Characterization of prostasin monoclonal antibodies

**Fig 4:**
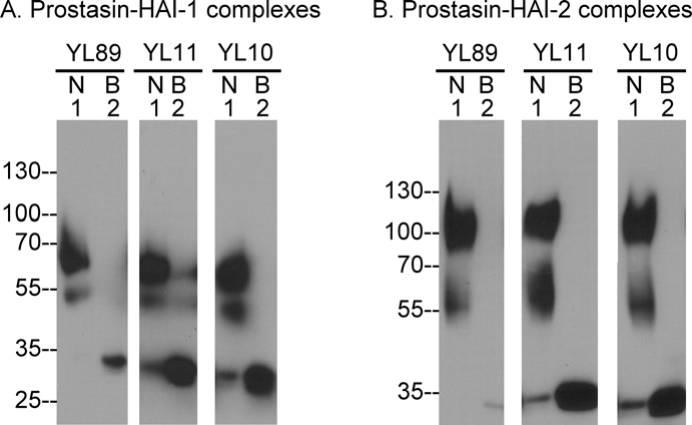
Characterization of prostasin monoclonal antibodies. Three prostasin monoclonal antibodies were generated using purified prostasin-HAI-1 complex as the antigen. The proteins eluted from HAI-1 mAb M19-Sepharose (A.) or HAI-2 mAb DC16-Sepharose (B.) immunoaffinity columns were analyzed by immunoblot using the three prostasin mAbs, YL11, YL10, and YL89 under non-boiled, non-reducing conditions (lanes 1) or boiled, non-reducing conditions (lanes 2).

In order to further characterize the zymogen activation and inhibition of prostasin by HAI-1 and HAI-2, we set out to generate prostasin monoclonal antibodies (mAbs) using purified, milk-derived prostasin-HAI-1 complexes to immunize mice, followed by conventional hybridoma technology. Using this approach we generated multiple monoclonal antibodies against prostasin, of which we selected three for further characterization: YL10, YL11, and YL89. These three prostasin mAbs all detect purified prostaisn-HAI-1 complexes (Fig 4A, lanes 1). The ratio of the 60-kDa relative to the 50-kDa complex detected by the three prostasin mAbs is similar to that seen in the Western blot analysis using HAI-1 mAb (Fig 2A) and with Protoblue staining of the proteins (Fig 2B, lane 1). The three mAbs all detected a 30-kDa protein bands upon heat treatment of the prostain-HAI-1 complexes (Fig 4A lanes 2), consistent with the thermal dissociation of the complex, as we observed in the diagonal gel electrophoresis (Fig 2C).

The three prostasin mAbs can also detect the two purified HAI-2 complexes using sample that have not been heat treated, and in the absence of reducing agent (Fig 4B, lanes 1) and the 30-kDa prostasin dissociated from the complexes after heat treatment (Fig 4B, lanes 2). It is worth noting that the heat treatment causes a significant reduction in the prostasin signal and so much more prostain-HAI-1 and prostasin HAI-2 complexes were needed for the Western blot analysis. The epitope recognized by the YL89 mAb appears to be even more sensitive to heat treatment than that recognized by mAbs YL10 and YL11.

### Human mammary epithelial cells are the likely in vivo cellular source for the matriptase and prostasin HAI-1 and -2 complexes

**Fig 5:**
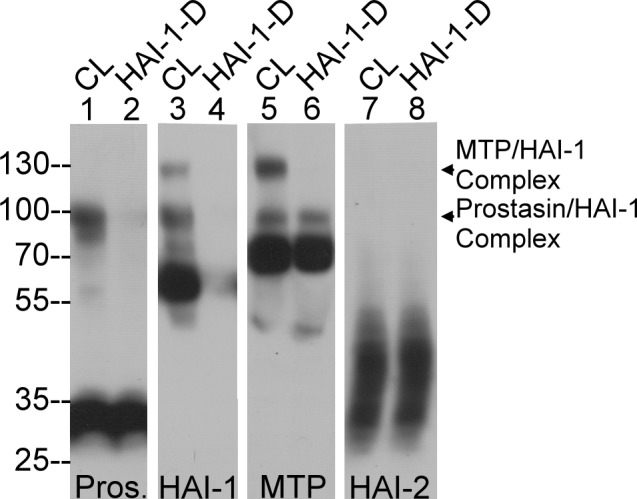
Analysis of prostasin, matriptase, HAI-1 and HAI-2 species expressed by human mammary epithelial cells. Lysates prepared from MTSV 1.7 milk-derived human mammary epithelial cells were analyzed by immunoblot for prostasin (Pros.), HAI-1, matriptase (MTP), and HAI-2 containing species before (lanes CL) and after (lanes HAI-1-D) immunodepletion of HAI-1 species from the lysates using HAI-1 mAb M19-Sepharose.

**Fig 6:**
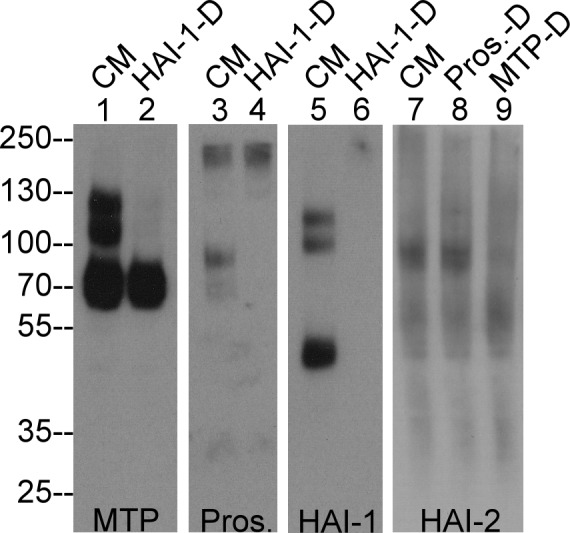
Human mammary epithelial cells secrete prostasin and matriptase as complexes with the HAIs. Conditioned medium collected from MTSV 1.7 milk-derived human mammary epithelial cells was analyzed by immunoblot for species containing matriptase (MTP), prostasin (Pros.), and HAI-1 before (lanes CM) and after (lanes HAI-1-D) immunodepletion of HAI-1 containing species with HAI-1 mAb M19-Sepharose. The conditioned medium was also analyzed by immunoblot for HAI-2 species after immunodepletion of prostasin (Pros.-D) or matriptase (MTP-D).

Most milk proteins are synthesized and secreted by the mammary epithelial cells. The expression of prostasin, matriptase, HAI-1, HAI-2, and their complexes were examined in the human mammary epithelial line MTSV 1.7, which was originally isolated and immortalized from human breast milk [15]. The four proteins were detected in cell lysate (Fig 5) and the conditioned medium from the cells (Fig 6). In cell lysates, prostasin was detected as its monomer of 33-kDa and in complex with HAI-1 around100-kDa (Fig 5, lane 1). The 100-kDa prostasin-HAI-1 complex was also detected by Western blot (Fig 5, lane 3) and its identity confirmed by its loss after immunodepletion using the HAI-1 mAb (Fig 5, lane 2). Matriptase was detected in its 70-kDa zymogen form and as the 120-kDa complex with HAI-1 (Fig 5, lane 5). Like prostasin-HAI-1 complex, the 120-kDa matriptase-HAI-1 complex was also detected by Western blot (Fig 5, lane 3) and removed by immunodepletion with the HAI-1 mAb (Fig 5, lane 6). HAI-2 was detected predominantly in its free form (Fig 5, lanes 7 and 8).

The expression status of these four proteins was next examined in the conditioned medium from the human mammary epithelial cells (Fig 6). Both matriptase-HAI-1 and prostasin-HAI-1 complexes were detected by Western blot and verified by immunodepletion. These shed complexes are smaller than their cell-associated counterparts, suggesting the involvement of proteolytic cleavage for their shedding into the extracellular milieu. Matriptase was also detected in its zymogen form (Fig 6, lane 1) and HAI-1 in its free form (Fig 6, lane 5) in the conditioned medium. While the levels of the HAI-2 species appear very low in the conditioned medium, a 90-kDa species was clearly detected (Fig 6, lane 7). The HAI-2 species can be immunodepleted by the matriptase mAb (Fig 6 lane 9) but not a prostasin mAb (Fig 6 lane 8), suggesting that this HAI-2 species contains matriptase.

## Discussion

**Fig 7:**
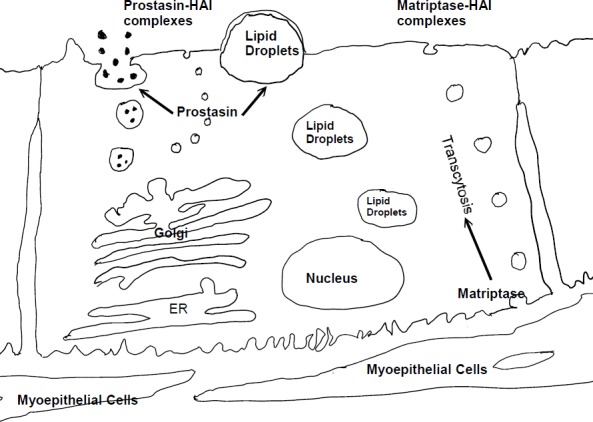
A schematic illustration for putative roles of matriptase and prostasin in lactating mammary gland.

The lactating mammary gland features 5 prominent processes relating to milk synthesis and secretion, including exocytosis, lipid synthesis and secretion, transcytosis of interstitial molecules, transport across the apical membrane, and the paracellular pathway [17]. The significant amounts of activated matriptase and activated prostasin found in milk suggest that matriptase and prostasin mediated proteolysis could contribute to lactation through one or more of the 5 processes involved in milk synthesis and secretion. Although the underlying mechanisms remain elusive, the subcellular localization of matriptase, prostasin, HAI-1 and HAI-2 provide hints as to the potential functional role of matriptase and prostasin proteolysis during lactation. Using Caco-2 human enterocytes as a model system of polarized epithelial cells, matriptase is targeted to the basolateral plasma membrane [16,18], where matriptase zymogen activation and inhibition by HAI-1 occur. Interestingly, matriptase-HAI-1 complex but not matriptase zymogen is secreted from the apical plasma membrane, indicating that a transcytosis process is very likely involved in the secretion of matriptase-HAI-1 complex from the apical surface of cells [16]. The basolateral localization and the transcytosis of (activated) matriptase-HAI-1 complex indicates that the basolateral plasma membrane is likely the functional location for matriptase and, suggests that matriptase may be involved in the transcytosis of interstitial molecules into the milk. As a GPI-anchored protein, prostasin may be targeted to the apical plasma membrane and subsequently internalized and then transported to early endosomal compartment [19] whereas in Caco-2 human enterocytes prostasin could be targeted to the apical plasma membrane via transcytosis and not internalized from the apical plasma membrane [18]. Prostasin might therefore potentially be involved in exocytosis, lipid synthesis and secretion, and transport across the apical membrane. The presence of prostasin in the higher-order HAI-2 complexes, which may be associated with casein micelles, is intriguing and suggestive of a role for prostasin in the secretory pathway and the exocytosis. Since prostasin is known to be involved in epidermal barrier function [20], which requires appropriately regulated lipid metabolism and secretion, prostasin could also be involved in the regulation of lipid synthesis and secretion during lactation. In Fig 7, we summarize the potential roles of matriptase and prostasin in lactating mammary gland.

Although the cultured human mammary epithelial cells express both HAI-1 and HAI-2, HAI-1 is the predominant inhibitor of matriptase and prostasin in these cells (Fig 5). The lack of prostasin-HAI-2 complexes detected in the cultured mammary epithelial cells may simply indicate that the mammary epithelial cells are not the *in vivo* source of the prostasin-HAI complexes detected in milk. Alternatively, these immortal milk-derived human mammary epithelial cells may have lost some *in vivo* programs involved in lactation responsible for translocating HAI-2 into the vicinity of prostasin thereby allowing the protease inhibitor to inhibit prostasin. In spite of the shared overall protein domain structure with a C-terminal transmembrane domain, HAI-2 has been detected primarily inside the cells not only in cultured mammary cells but also *in vivo* in colon epithelial cells [13,21,22]. In contrast, HAI-1 is detected at cell-cell junctions where HAI-1 is co-localized with matriptase [23]. Alterations in subcellular localization of HAI-2 has been observed in breast cancer cells in which inhibition of matriptase by HAI-2 has been observed [13]. It is reasonable to hypothesize that lactating mammary epithelial cells *in vivo* utilize these membrane-bound serine proteases and serine protease inhibitors for milk synthesis and secretion along with the significant remodeling of cellular structure involved in lactation. The selective inhibition of matriptase by HAI-2 could be, in part, the reason that doxycycline-induced HAI-2 is unable to suppress the activation of the protease activated receptor (PAR)-2 by matriptase co-expressed in HEK293 cells [24]. HAI-2 seems able to significantly suppress PAR-2 activation when matriptase and prostasin are co-expressed. It remains unclear, however, whether matriptase proteolytic activity is involved in the PAR-2 activation observed in this context because matriptase zymogen activation is apparently negligible in HEK293 cells modified to overexpress prostasin, matriptase and HAI-2 [24]. The functional relationship between matriptase and HAI-2 might go beyond the simple interaction between an active serine protease and a Kunitz domain. HAI-2 was reported to bind to full-length matriptase (zymogen form), in which the N-terminal processing via the autolytic cleavage of Gly-149 within the SEA domain has not occurred. HAI-2 was also reported to interact, though at lower affinity, with the mature matriptase zymogen form, in which SEA domain cleavage has occurred [24]. Protease inhibitors typically only exhibit strong binding affinity with the active form of a serine protease and not the zymogen form [25], and so the binding between HAI-2 and matriptase zymogen reported by Friis et al is most likely some unconventional type of interaction between protease and protease inhibitor. It remains unclear what the structural basis for an interaction between matriptase zymogen and HAI-2 might be, nor the difference in the HAI-2 binding affinity to full-length matriptase zymogen *versus* mature matriptase zymogen. As the studies were conducted using cells modified to induce the expression of these proteins, it seems possible that the interactions observed might be the result of the ectopic expression of the proteins at atypical levels or in unusual sub-cellular compartments.

The most striking difference between HAI-1 complexes and HAI-2 complexes in human milk is that HAI-1 complexes readily bind to protein purification columns whereas the majority of HAI-2 complexes with prostasin do not. One possibility for their lack of binding to purification columns is that the majority of HAI-2 complexes are in fact part of high-order complexes, which prevents HAI-2 complexes from binding to either cation or anion exchangers, (DEAE and CM Sepharoses) and to the immunoaffinity columns. A small proportion of the total HAI-2 complexes are released from the higher-order complexes and so can be purified by chromatography columns. One example of high-order protein complexes that are present in milk are the casein micelles. Based on thermodynamic studies, it is known that hydrophobic effects and main-chain hydrogen bonding can drive kappa-casein micelle self-assembly [26]. The variability and plasticity of casein micelle structure is a direct consequence of its amorphous, unfolded idiosyncratic nature, with the result that it is capable of binding to a wide range of target proteins. If the vast majority of HAI-2 complexes are incorporated into casein micelles, it may be that HAI-2 and its target proteases contribute to lactation via the synthesis or processing of casein micelles. Furthermore, as a highly glycosylated protein, the carbohydrates added post-translationally to HAI-2 are capable of donating and accepting large quantities of hydrogen bonds. It is therefore conceivable that kappa-casein molecules form micelles around the HAI-2 complexes.

The presence of matriptase and prostasin in complexes with HAI-1 and HAI-2 in human milk suggests that matriptase and prostasin are the physiologically relevant target proteases of HAI-1 and HAI-2 in this system. Because there was no uncomplexed HAI-1 or HAI-2 detected in milk (Fig 1), it seems likely that the secretion of these complexes is a highly selective process, which does not allow uncomplexed HAI-1 or HAI-2 to be secreted. Furthermore, the formation of these protease-protease inhibitor complexes most likely occurs prior to their secretion with the proteases and proteases inhibitor being subsequently secreted as tightly associated complexes. It is very unlikely that these protease-protease inhibitor complexes form as a simple biochemical interaction occurring in the milk after the proteases and protease inhibitors are separately secreted as individual molecules. This is particularly true for the matriptase-HAI-1 complex, which is formed in the basolateral plasma membrane and secreted from apical surface [16]. In addition to the mammary epithelial cells, the lactating mammary gland contains many other cell types, which could also contribute to the secretion of the HAI-1 and HAI-2 complexes detected in milk. For example, matriptase is expressed by some monocytes/macrophages, in which HAI-1 is either not expressed, or is expressed at very low levels. It will be interesting to determine if HAI-2 can replace and/or function in concert with HAI-1 in the control of matriptase in monocytes/macrophages.

Prostasin has been suggested to play a role in matriptase zymogen activation through the formation of a reported reciprocal zymogen activation complex [27], which implies the presence of matriptase-prostasin-HAI-1 complexes. The prostasin-HAI-1 complex isolated from milk in this study, however, definitely contains no matriptase. In Fig 1A (lanes 6 and 7), the matriptase mAb did not detect the prostasin-HAI-1 complex. Furthermore, there was no matriptase detected in our protein identification study in Fig 2. The diagonal gel electrophoresis study also reveals that the complex contains only prostasin and HAI-1, based on their sizes and the stoichiometry of one prostasin with one HAI-1. Protein-protein interactions take place with various binding strength from the strongest ones mediated through covalent bond, to ones with very tight non-covalent binding such as in Ab-Ag complexes and protease-protease inhibitor complexes, to ones with relatively weak interaction which nevertheless can be detected using immunoprecipitation followed by immunoblot. The non-covalent interactions between serine proteases and Kunitz inhibitors are strong and can survive SDS-PAGE under non-boiled and non-reducing conditions [1,28]. The reciprocal zymogen activation complex proposed by Friis et al would be expected to involve relatively weak interactions and to have a relatively transient existence. In the current study, the interaction between the two proteases and the two inhibitors are very strong and long-lasting. These complexes survive exposure to 1% SDS and thus can be detected by Western blot. The complexes proposed in the Friis studies were isolated by immunoprecipitation followed by identification of the constituents by immunoblot. If matriptase-prostasin-HAI complexes do in fact exist, they would not survive the conditions used in this work and so are very different from the complexes described in the current study.

In summary, the presence of activated matriptase and prostasin in complexes with HAI-1 and HAI-2 in human milk suggests that the membrane-associated serine proteases matriptase and prostasin are significantly active but under tight control during lactation. Although if and how matriptase and prostasin contribute to lactation remains unclear, HAI-1 and HAI-2 represent the most prominent protease inhibitors responsible for the control of matriptase and prostasin mediated proteolysis during lactation. Our study sheds light on novel mechanisms that provide a link between cell surface proteolysis and offspring nutrition and immunity.
